# Prothrombin Time, Activated Partial Thromboplastin Time, and Fibrinogen Reference Intervals for Inbred Strain 13/N Guinea Pigs (*Cavia porcellus*) and Validation of Low Volume Sample Analysis

**DOI:** 10.3390/microorganisms8081127

**Published:** 2020-07-27

**Authors:** Jillian A. Condrey, Timothy Flietstra, Kaitlyn M. Nestor, Elizabeth L. Schlosser, JoAnn D. Coleman-McCray, Sarah C. Genzer, Stephen R. Welch, Jessica R. Spengler

**Affiliations:** 1 Comparative Medicine Branch, Division of Scientific Resources, Centers for Disease Control and Prevention, Atlanta, GA 30329, USA; nro8@cdc.gov (J.A.C.); opn0@cdc.gov (K.M.N.); pii8@cdc.gov (E.L.S.); muz8@cdc.gov (S.C.G.); 2 Viral Special Pathogens Branch, Division of High-Consequence Pathogens and Pathology, Centers for Disease Control and Prevention, Atlanta, GA 30329, USA; hng8@cdc.gov (T.F.); flj7@cdc.gov (J.D.C.-M.); yos6@cdc.gov (S.R.W.)

**Keywords:** coagulation, clotting, hemorrhagic fever, animal model, guinea pig, strain 13/N, *Cavia porcellus*, prothrombin time, activated partial thromboplastin time, fibrinogen, VetScan VS*pro*

## Abstract

Inbred strain 13/N guinea pigs are used as small animal models for the study of hemorrhagic fever viruses. Coagulation abnormalities, including prolonged clotting times and bleeding, are characteristic of hemorrhagic fever in humans; patients often meet criteria for disseminated intravascular coagulation (DIC). Comprehensively evaluating coagulation function is critical in model development and studies of viral pathogenesis and therapeutic efficacy. Here, using the VetScan VS*pro* veterinary point-of-care platform, we developed reference intervals in both juvenile and adult strain 13/N guinea pigs for three coagulation parameters: prothrombin time (PT), activated partial thromboplastin time (aPTT), and fibrinogen. In addition, for situations or species with limited availability of blood for clinical analysis, we investigated the validity of a modified collection approach for low-volume (0.1 mL) blood sample analysis of PT and aPTT.

## 1. Introduction

In severe viral hemorrhagic fever (VHF) disease, coagulation abnormalities driven by robust inflammatory responses in the body are thought to be a cause of death in human patients [1]. Many VHF agents, including Ebola [2,3], Marburg [4], and Crimean-Congo hemorrhagic fever viruses [5], target macrophages and other circulating immune cells, resulting in cytokine production. Cytokine activity promotes vascular leakage and hypotension and can activate coagulation pathways that may ultimately lead to disseminated intravascular coagulation (DIC) [6]. In human patients with Lassa fever, for example, abnormalities include prolonged prothrombin time (PT), prolonged whole blood clotting time, and isolated thrombocytopenia [7].

When small blood vessels or tissues are damaged, as may occur in VHF infection, the body controls this damage via physiological processes referred to as hemostasis. Hemostasis depends on an interaction between the plasma-based coagulation cascade, platelets, and the endothelium of blood vessels. Clinical laboratory analytical assays can measure only the first two components of this system. In particular, coagulation tests, such as PT, activated partial thromboplastin time (aPTT), and thrombin time, are used to assess blood clotting function in patients. Levels of fibrinogen, a soluble protein in the plasma that is enzymatically broken down to fibrin to form clots, may also be evaluated. These laboratory tests may be helpful in elucidating the cause of unexplained bleeding or identifying abnormalities in clotting function due to disease.

Inbred strain 13/N guinea pigs are an important small animal model for studying highly pathogenic human viruses, including arenaviruses. Arenaviruses are the causative agents of severe and frequently fatal human hemorrhagic fevers like Lassa fever and Lujo hemorrhagic fever. Strain 13/N guinea pigs can be readily infected with Lassa and Lujo viruses without serial host adaption required by other animal models, such as mice and outbred guinea pigs [8]. These guinea pigs have been used to investigate viral pathogenesis [9,10,11,12], screen therapeutics [13], and develop vaccines [14,15,16,17,18] for arenaviruses and other high-consequence agents. Despite frequent use in studies of VHFs, to date, no normal reference ranges for coagulation factors have been reported in strain 13/N guinea pigs. To aid with investigations using this model, we aimed to define reference ranges for three parameters of clotting function in strain 13/N guinea pigs: PT, aPTT, and fibrinogen. Furthermore, we hypothesized that a novel blood collection method performed in accordance with recommended anticoagulant-to-sample ratio has consistent PT and aPTT values regardless of sample volume. Thus, to expand applications of the VetScan VS*pro* platform for use in smaller species or in situations of limited blood sample volume, we also assessed the validity of a new method for low volume sodium citrate blood collection (0.1 mL vs. recommended 0.5 mL) for PT and aPTT analysis.

## 2. Materials and Methods

### 2.1. Ethics Statement

Animal work was approved by the Centers for Disease Control and Prevention (CDC) Institutional Animal Care and Use Committee (IACUC) and conducted in accordance with the Guide for the Care and Use of Laboratory Animals, 8th Edition, at an AAALAC-accredited facility.

### 2.2. Animals

Healthy strain 13/N guinea pigs were selected from the CDC breeding colony for establishing coagulation reference intervals and validation of low volume blood sample analysis of PT/aPTT. Age groups were defined as 0–150 days old for juveniles and 151–900 days old for adults. As previously described [19], cut-offs for juveniles and adults in the colony were determined based on the established age of sexual maturity, onset of decreased fecundity, and development of age-related diseases [20,21]. Sows with pups younger than 6 weeks were housed in conventional rack-style caging (Techniplast, West Chester, PA, USA). Active breeding groups were housed in rack-style caging or in floor pens. All other animals were socially housed in sex-segregated floor pens with paper (Techboard and Poly Pads, Shepherd Specialty Papers, Watertown, NY, USA) and paper nesting material (EnviroDri, FiberCore LLC, Cleveland, OH, USA). Guinea pigs were provided unrestricted bottled spring water (Niagara Bottling, Ontario, CA, USA) and pelleted diet (LabDiet 5025, Land O’Lakes, St. Louis, MO, USA) with daily timothy hay and supplemental vitamin C-rich fresh vegetables three times weekly. Environmental enrichment was provided in the form of plastic tunnels (Guinea Pig Hut, Bio-Serv, Flemington, NJ, USA) and wood gnawing materials (manzanita sticks and wood blocks, Bio-Serv, Flemington, NJ, USA). Environmental parameters were maintained within a temperature range of 68–79°F and 30–70% relative humidity on a 12:12 h light:dark cycle. Annual health monitoring of the colony included serology (Guinea Pig Basic Opti-Spot, IDEXX BioResearch, Columbia, MO, USA), baseline complete blood counts, and clinical chemistry analyses on a subset of animals. All evaluated animals were free of *Clostridium piliforme*, guinea pig parainfluenza virus 3, lymphocytic choriomeningitis virus, murine pneumonia virus, Sendai virus and *Encephalitozoon cuniculi*, as determined using serological screening tests [19].

### 2.3. Blood Collection

Blood samples (0.6 mL, not to exceed 7.5% of total blood volume) were collected from the cranial vena cava with a 5/8-inch-long 25 gauge needle and 1 mL syringe (Becton-Dickinson, Franklin Lakes, NJ, USA); during collection, animals were anesthetized using 3–5% isoflurane administered via a facemask. A 0.1 mL micropipette (Sarstedt Inc. 17.2111.100, Nümbrecht, Germany) was used to exactly measure 0.1 mL of blood directly from the syringe. Using the micropipette, blood was transferred to a 0.5 mL sterile screw cap micro tube (Sarstedt Inc. 72.730.105) pre-filled in-house with 11 µL of 3.2% sodium citrate (ANIARA A12-8480-10, West Chester, OH, USA). The remaining 0.5 mL of blood in the syringe was transferred into a 0.5 mL 3.2% sodium citrate tube (Sarstedt Inc. 41.1506.102). Multiple samples could be taken from the same animal if > 7 days after the previous blood draw. All samples were obtained between 10:00 a.m. and 3:00 p.m. to limit impact of natural daily fluctuations. Samples displaying visible clots were not included in the data set. All coagulation tests were run within 2 h of sampling.

### 2.4. PT/aPTT

PT/aPTT was measured using the VetScan VS*pro* and Coagulation Cartridge (Abaxis Inc., Union City, CA, USA) using 60 µL of blood directly from the sodium citrate tube. All blood samples were gently inverted 10 times every 5–10 min to prevent clot formation in the tubes and immediately prior to pipetting into the PT/aPTT cartridges. The PT/aPTT measurements were recorded along with corresponding sample collection size (0.1 vs. 0.5 mL).

### 2.5. Fibrinogen

Fibrinogen was measured using the VetScan VS*pro* and Fibrinogen Test Cartridge (Abaxis Inc., Union City, CA, USA). All VS*pro* machines were calibrated using the Fibrinogen Calibration Test Kit (Abaxis Inc., Union City, CA, USA). After running the PT/aPTT from the 0.5 mL sodium citrate tube, the remaining sample was centrifuged for three min at 4000× *g* and 0.1 mL of plasma was transferred to the diluent tube (supplied with fibrinogen test cartridge). Using a pipette, the diluent and sample were mixed thoroughly. Once prompted by the VS*pro*, 0.1 mL of the mixture was transferred onto the test cartridge and the measurement was recorded.

### 2.6. Statistical Analyses

Data were analyzed using Microsoft Access (v. 365). ANOVA was used to determine the effect of age and sex on the averages for the three analytes. Paired *t*-tests were used to determine the differences between the 0.1 and 0.5 mL samples for PT and aPTT. Additionally, kernel density estimation (KDE) was used to compare the distributions of the two sampling volumes. A *p*-value < 0.05 was considered statistically significant in this study. Samples from one adult (T181925) were excluded from the KDE for aPTT because they were identified as outliers that were biasing the analysis, by skewing the density function.

## 3. Results

### 3.1. Reference Intervals for Strain 13/N Guinea Pigs

Both PT and aPTT are measures of the function of the common coagulation pathway; in addition, PT is a measure of the extrinsic pathway, whereas aPTT is a measure of intrinsic pathway. PT is the time in seconds that it takes plasma to clot after the addition of phospholipid, tissue factor (factor III), and calcium to the specimen. aPTT is the time in seconds that it takes plasma to clot after the addition of a contact agent that fully activates factors XII along with calcium and phospholipids. When differentiating PT from aPTT, “partial” in aPTT refers to the addition of phospholipid (but not tissue factor) and “activated” refers to the contact activators used to accelerate fibrin formation in the assay [22]. PT or aPTT values that are higher than average are considered clinically relevant, and should be interpreted in conjunction with other analytes to identify a clotting abnormality, e.g., fibrinogen, platelet count, or thrombin time. In general, a clotting abnormality is evident if a PT or aPTT value is >1.5× above the upper end of the reference interval.

The VetScan VS*pro* is a point-of-care platform offering a rapid quantitative PT/aPTT combination test to perform both assays in parallel using a microcapillary design that aspirates citrated whole blood from a reservoir. The blood, traveling through two parallel capillary paths, is exposed to activators for the respective coagulation tests and then flows into a labyrinth of capillaries that cross an aperture for a light. The light detects the time at which the capillary blood flow stops, which is the test end point [22]. The test dynamic ranges for PT and aPTT measurements on the VS*pro* are 11–35 and 30–200 s, respectively. Fibrinogen is an acute phase protein made in the liver; concentrations in blood may increase due to an inflammatory response or decrease due to consumption (e.g., DIC). Fibrinogen has a fairly wide range depending on species, but is generally 1–4 g/L [23,24]. Fibrinogen levels outside of the reference interval indicate either excessive consumption, or release due to acute phase response to inflammation. The VetScan VS*pro* fibrinogen test cartridge is a quantitative test marketed for testing in equine platelet-poor plasma from a citrate-stabilized whole blood sample.

**Figure 1:**
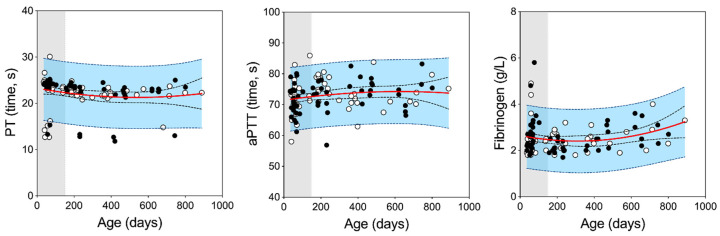
Prothrombin time, activated partial thromboplastin time and fibrinogen values for juvenile and adult strain 13/N guinea pigs. Sodium citrate whole blood samples collected from juvenile (shaded; age ≤150 days) and adult (unshaded; age 151–900 days) were processed and analyzed for PT, aPTT, and fibrinogen. Open circles, female; closed circles, male; red line, mean; black dotted lines, confidence intervals of the mean; dotted blue lines and blue shading, 95% predictive values.

To evaluate parameters of clotting function (PT, aPTT, and fibrinogen) for strain 13/N guinea pigs, a total of 111 samples were analyzed from 109 animals; two samples were repeat samples collected from the same individual: the first during the juvenile period and again during adulthood (Figure 1). Samples were obtained from 52 juveniles (age range 36–140 days; 25 females and 27 males) and 59 adults (age range 157–889 days; 28 females and 31 males). Not all analyses were completed on every animal due to cartridge/machine error or presence of hemolysis in fibrinogen plasma samples. Reference ranges were developed for each age group and further divided within the age group by sex for 0.5 mL whole blood sample collection and analysis per manufacturer recommendations (Table 1, Figure 1). An ANOVA was conducted to determine effects of age and sex on PT, aPTT, and fibrinogen; neither significantly affected PT (f-stat = 0.77, *p*-value = 0.46) or fibrinogen (f-stat = 1.5, *p*-value = 0.22). The ANOVA found there was a significant effect of these factors on aPTT (f-stat = 3.3, *p*-value = 0.04), with age as the only significant factor (t-stat = 2.27, *p*-value = 0.028) measuring an average aPTT for adults 2.17 s higher than juveniles.

### 3.2. Validation of the Low Volume Collection Approach

The VS*pro* PT/aPTT combination test requires a minimum of 60 µL of whole blood. However, 0.5 mL is the lowest volume of commercial sodium citrate blood collection tubes currently available. Total blood volume in smaller species may prohibit collection of adequate serial or terminal exsanguination samples for full coagulation analysis using the required anticoagulant-to-sample ratio in commercially available collection tubes (0.5 mL). Especially in small-volume collection tubes, under-filling may cause significant sample dilution and may provide falsely prolonged clotting times due to the excess calcium-binding citrate present [25,26]. Thus, we developed an in-house sample collection method to achieve equivalent anticoagulant-to-sample ratio for a 0.1 mL blood sample. Our in-house collection method was based on recommendations by the Clinical and Laboratory Standards Institute of a 1:9 ratio of 3.2% sodium citrate to blood for aPTT and PT coagulation studies in humans [27].

To evaluate our 0.1 mL sampling approach, we analyzed 108 PT samples (age range 36–889 days; 52 females and 56 males) and 106 aPTT samples (age range 36–889 days; 50 females and 56 males). We collected 0.5 and 0.1 mL volumes from an individual originating from the same collection syringe, i.e., a 0.1 mL aliquot of the sample ran parallel with a 0.5 mL aliquot (as described in Section 2). Statistical differences between data generated from paired high- and low-volume samples were evaluated by age and sex using a paired *t*-test. We found no statistical differences between the 0.1 and 0.5 mL samples for aPTT (*p*-value = 0.360), but did find a statistical difference between the paired PT samples (*p*-value = 0.049). Notably, this difference was only seen in samples from juvenile animals. The difference between PT sample volumes for blood collected from juveniles (age range 36–140 days; 24 females and 26 males) was significantly different (*p*-value = 0.012), whereas in blood collected from adults (age range 157–889 days; 28 females and 30 males), the difference between 0.1 and 0.5 mL samples was not significant (*p*-value = 0.588) (Table 2).

**Figure 2:**
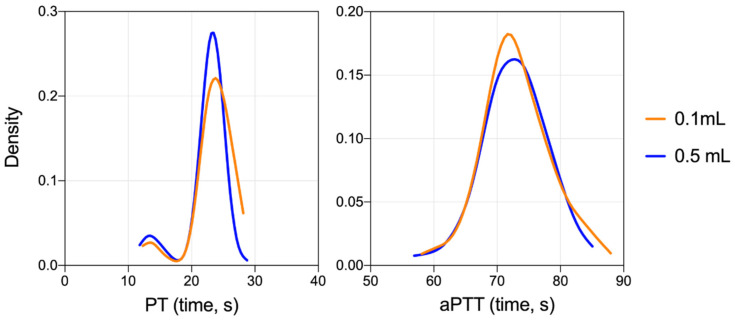
Kernel density estimation for prothrombin time and activated partial thromboplastin time from 0.1 mL or 0.5 mL samples. Values from both PT and aPTT tests were evaluated using the kernel density estimation (KDE), a non-parametric estimate of the probability density function of a random variable. The KDE demonstrated overlapping distribution between 0.1 mL and 0.5 mL collection volumes for both PT and aPTT.

To further examine this difference between sample collection volumes, we performed KDE for samples from PT and aPTT tests (Figure 2). We found that the distribution of values was similar between data obtained with both volume sizes; little variability was introduced by using 0.1 mL samples. The average difference between collection volumes was small (±1 s). Furthermore, in the PT test, both sample volumes revealed values appearing lower than the main distribution curve (i.e., at ~13–15 s), representing normal physiological variation in data.

## 4. Discussion

Assessments of PT, aPTT, and fibrinogen in small laboratory animal species are an important component in research of both infectious [28,29,30] and chronic diseases [31]. These analytes are of particular importance when modeling conditions in which identification of coagulation abnormalities aids in diagnosis or guides treatment. Guinea pigs are commonly used for modeling VHF disease, and coagulation values were reported in many of these studies [10,32,33,34,35]. Strain 13/N guinea pigs are used frequently in arenavirus research [10,36,37,38,39,40], but also to study other VHFs such as Ebola virus disease [41]. However, despite their frequent use in modeling, reference ranges for common clotting tests and indicators of clotting abnormalities in strain 13/N guinea pigs were not previously reported.

Normal PT, aPTT, and fibrinogen values were reported for both inbred and outbred guinea pig strains [42,43,44,45], as well as for other small laboratory animal species, including mice, rats, and hamsters [46,47,48]. In general, the reported ranges between species and between strains within species vary considerably, which could be attributed to physiological differences or differences in testing methodology. In particular, due to differences in test chemistry (e.g., contact activators) and readout, reference ranges are machine- and reagent-specific and are not interchangeable [22]. In addition, variation in clotting parameters may be associated with the strain and/or sex of the animal, sampling technique, or type of anesthesia used [46,49,50,51]. In our studies, values for strain 13/N guinea pigs did not significantly differ based on sex for any of the analytes. In addition, reference ranges were not significantly different for PT and fibrinogen based on age. However, aPTT values were significantly different between juvenile and adult groups, but only by an average of 2.17 s. Currently, two papers reported plasma PT and aPTT/PTT in guinea pigs; both of those papers found PT to be longer than aPTT or PTT [42,44]. Here, we found whole blood PT values to be shorter than aPTT, which is consistent with the majority of other reports in a wide range of species such as mice, rats, rabbits, dogs, sheep, cattle, and non-human primates [48,51,52].

Whereas many clinical assays are not functionally species-specific, point-of-care platforms are often not commercially validated for use in all species. For example, the processing of fibrinogen, an extracellular protein found in significant concentrations in the blood plasma of all vertebrates, is conserved between mammals [53,54,55]. The VS*pro* fibrinogen assay used here measures fibrinogen by thrombin-mediated enzymatic conversion to fibrin; thus, nothing precludes the use of this platform for other species. When our studies began, the assay was only validated for use in horses. However, studies are ongoing and the manufacturer has confirmed that the assay is now also validated for dogs. Additionally, although the VetScan VS*pro* PT/aPTT combination test is marketed for cats and dogs, it has previously been used to assess coagulation in guinea pig studies of arenavirus infection [33].

Reports of PT, aPTT, and fibrinogen in small laboratory animal species are based on a variety of approaches and platforms (e.g., VetScan VS*pro*, STart4 instrument, ACL Elite Pro), and often list a 0.5 mL collection volume. This volume is consistent with the smallest commercially available sodium citrate tube, which necessitates 0.5 mL of whole blood for proper mixing. The STart4 and ACL Elite Pro instruments require centrifugation of whole blood to form plasma, whereas the VetScan VS*pro* only requires whole blood, decreasing the overall sample amount needed for coagulation quantification. As collection volume is often not detailed in studies and since the effects of blood collection volume on coagulation values have not been reported, we expanded our studies to investigate this question.

For species or studies in which blood sample volume may be limited, we assessed a novel low-volume collection technique using the PT/aPTT assay on the VS*pro* platform. We found that collection of 0.1 mL following the methods described generated comparable values to paired 0.5 mL samples run in parallel. The only statistical difference was between PT samples from juveniles; this may be due to data artifacts or to normal variation in the assay, as coagulation assays are particularly sensitive to pre-analytical variables including those that may affect sample quality (e.g., hemolysis or micro clots) [56]. However, in this cohort, the average difference between the 0.1 and 0.5 mL sample collection volumes was only 1 s, which is well within 1 SD of our established reference range for PT, suggesting that this difference is not clinically relevant. Altogether, these data support our hypothesis and the utility of the method described here (using a premeasured micropipette to transfer sample to a corresponding in-house prepared sodium citrate tube) for low blood volume collection and analysis of PT and aPTT.

## Figures and Tables

**Table 1:** Prothrombin time, activated partial thromboplastin time and fibrinogen reference intervals for strain 13/N guinea pigs.

**Juvenile (Age ≤150 days)**								
**Analyte (unit)**	***n***	**Mean**	**SD**	**LRR**	**URR**	**Median**	**LIQ**	**UIQ**
**Female**								
PT (time, s)	25	22.66	4.30	14.24	31.09	23.6	23.1	24.7
aPTT (time, s)	23	72.03	4.94	62.35	81.70	72.1	70.2	74.7
Fibrinogen (g/L)	25	2.45	0.78	0.93	3.97	2.3	1.9	2.5
**Male**								
PT (time, s)	26	23.07	3.07	17.05	29.10	24.1	23.5	24.2
aPTT (time, s)	26	70.71	4.96	60.98	80.44	69.8	67.3	74.2
Fibrinogen (g/L)	27	2.75	0.88	1.03	4.47	2.6	2.2	3.1
**Adult (Age 151–900 days)**								
**Analyte (unit)**	***n***	**Mean**	**SD**	**LRR**	**URR**	**Median**	**LIQ**	**UIQ**
**Female**								
PT (time, s)	28	22.10	1.69	18.79	25.42	22.3	21.7	23.0
aPTT (time, s)	28	74.16	4.67	65.01	83.31	73.7	71.0	78.9
Fibrinogen (g/L)	27	2.52	0.53	1.49	3.55	2.4	2.1	2.9
**Male**								
PT (time, s)	31	21.44	3.97	13.65	29.22	22.9	21.9	23.5
aPTT (time, s)	31	73.50	5.18	63.34	83.66	74.5	70.2	76.6
Fibrinogen (g/L)	28	2.46	0.51	1.47	3.46	2.4	2.1	2.8

aPTT, activated partial thromboplastin time; *n*, sample number; PT, prothrombin time; s, seconds; SD, standard deviation; LRR, lower reference range; URR, upper reference range; LIQ, lower interquartile range; UIQ, upper interquartile range.

**Table 2:** Paired *t*-test results for 0.1 vs. 0.5 mL sample volume values for prothrombin time and activated partial thromboplastin time in seconds for all animals, or by age or gender.

Variable	Group	Mean Diff	SD Diff	*n*	*t*-Stat	*p*-Value
**PT**	All	0.99	4.56	108	2.26	0.026
	Juveniles	1.79	4.83	50	2.62	0.012
	Adults	0.30	4.24	58	0.55	0.588
	Females	0.66	4.23	52	1.12	0.269
	Males	1.30	4.87	56	2.00	0.050
**aPTT**	All	0.82	9.18	106	0.92	0.360
	Juveniles	2.16	6.60	48	2.27	0.028
	Adults	−0.29	10.80	58	−0.21	0.838
	Females	0.32	6.51	50	0.35	0.728
	Males	1.26	11.08	56	0.85	0.397

Mean Diff, mean difference; SD Diff, standard deviation difference; *n*, sample number; *t*-Stat, *t*-statistic; *p*-Value, probability value. Age groups defined as juveniles (age ≤150 days) or adults (age 151–900 days).
